# EuGI: a novel resource for studying genomic islands to facilitate horizontal gene transfer detection in eukaryotes

**DOI:** 10.1186/s12864-018-4724-8

**Published:** 2018-05-03

**Authors:** Frederick Johannes Clasen, Rian Ewald Pierneef, Bernard Slippers, Oleg Reva

**Affiliations:** 1 Centre for Bioinformatics and Computational Biology; Department of Biochemistry, Genetics and Microbiology, University of Pretoria, Pretoria 0002, Private Bag X20, Hatfield, 0028 South Africa; 2 Forestry and Agricultural Biotechnology Institute; Department of Biochemistry , Genetics and Microbiology, University of Pretoria, Pretoria, 0002 South Africa

**Keywords:** Genomic island, Horizontal gene transfer, SWGIS v2.0, Eukaryotes, Comparative genomics, Software tools

## Abstract

**Background:**

Genomic islands (GIs) are inserts of foreign DNA that have potentially arisen through horizontal gene transfer (HGT). There are evidences that GIs can contribute significantly to the evolution of prokaryotes. The acquisition of GIs through HGT in eukaryotes has, however, been largely unexplored. In this study, the previously developed GI prediction tool, SeqWord Gene Island Sniffer (SWGIS), is modified to predict GIs in eukaryotic chromosomes. Artificial simulations are used to estimate ratios of predicting false positive and false negative GIs by inserting GIs into different test chromosomes and performing the SWGIS v2.0 algorithm. Using SWGIS v2.0, GIs are then identified in 36 fungal, 22 protozoan and 8 invertebrate genomes.

**Results:**

SWGIS v2.0 predicts GIs in large eukaryotic chromosomes based on the atypical nucleotide composition of these regions. Averages for predicting false negative and false positive GIs were 20.1% and 11.01% respectively. A total of 10,550 GIs were identified in 66 eukaryotic species with 5299 of these GIs coding for at least one functional protein. The EuGI web-resource, freely accessible at http://eugi.bi.up.ac.za, was developed that allows browsing the database created from identified GIs and genes within GIs through an interactive and visual interface.

**Conclusions:**

SWGIS v2.0 along with the EuGI database, which houses GIs identified in 66 different eukaryotic species, and the EuGI web-resource, provide the first comprehensive resource for studying HGT in eukaryotes.

**Electronic supplementary material:**

The online version of this article (10.1186/s12864-018-4724-8) contains supplementary material, which is available to authorized users.

## Background

Genomic islands (GIs) are genomic fragments that resulted from the insertion of mobile genetic elements and have been shown to facilitate the evolutionary processes in bacteria and archaea [1, 2]. Horizontal gene transfer (HGT) allows organisms to rapidly adapt to fluctuating environments and different ecological pressures or opportunities [3, 4]. Studies of bacterial pan-genomes suggest that microbial genomes continuously harvest new genetic information through HGT from a pool of genetic material available as “free goods” and that the accessory genome contributes significantly to bacterial niche speciation [5]. The acquisition of GIs have also been shown to be central in the evolution of pathogenesis in virulent microbes [6].

Research of HGT in prokaryotes far exceeds that of eukaryotes. There is, however, compelling examples in some eukaryotic species of where HGT allowed ecological niche speciality [7, 8], drove metabolic innovation and expansion [9, 10] and accelerated the adaptation to completely novel lifestyles [11, 12]. Albeit less frequent than in prokaryotes, horizontally transferred genes in eukaryotic genomes can clearly also provide an important selective advantage for these species. The impact of HGT on all domains of life are therefore becoming increasingly evident [13].

Methods of GI detection is broadly divided into those using a parametric (or surrogate) or a comparative approach [14]. Parametric methods inherently rely on the comparison of the sequence composition of GIs to that of the surrounding genome or to the average sequence composition of the genome. In contrast, comparative methods rely on identification of incongruence in taxonomic relations between organisms and phylogenetic inferences based on selected chromosomal loci [4, 14, 15]. Both parametric and comparative methods have been shown to have advantages and disadvantages. Both produce false positives and false negative errors [16–19], especially between closely related taxa, in weakly sampled taxonomic lineages and for genes that evolve at unusual rates [14, 20]. Using both approaches to complement each other is therefore preferable.

Studies of HGT in eukaryotes to date focussed on identifying horizontally transferred genes with comparative methods rather than the identification of inserts of GIs with parametric methods. Potentially transferred genes were detected either through exhaustive BLAST searches to identify genes with a higher sequence similarity to bacterial homologs compared to eukaryotic homologs [10, 21], or by phylogenetic incongruence between gene and species trees [9, 22]. Exploiting the sequence composition bias of GIs with parametric methods commonly used in prokaryotes, is consequently limited in eukaryotes [23, 24]. A possible reason for this is that parametric methods can be computationally intensive on large eukaryotic genomes and that large heterogeneous chromosomes can lead to high rates of false positive identification.

The main objective of this study is to test and modify the algorithm of the prokaryotic GI search tool, SWGIS, as a rapid tool for initial identification of potential GIs in eukaryotic chromosomes. The modified search tool, SWGIS v2.0, is then used to predict GIs in the genomes of a representative sample of fungal, protozoan and invertebrate species in order to determine how common such events are across this range of eukaryotes. Finally, we aim to develop a database and associated search tools of eukaryotic GIs to aid in eukaryotic HGT studies, similar to what exists for prokaryotes.

## Implementation

### Identification of genomic islands

SeqWord Gene Island Sniffer (SWGIS) algorithm [19] was modified to SWGIS v2.0 and used for prediction of GI locations in chromosomal sequences. Oligonucleotide usage pattern (OUP) was denoted as a matrix of deviations Δ_[ξ1…ξ*N*]_ of observed over expected counts of all possible tetranucleotide permutations:

1 $$ {\varDelta}_{\left[{\xi}_1\dots {\xi}_N\right]}=\left({C}_{\left[{\xi}_1\dots {\xi}_N\right]\mid obs}-{C}_{\left[{\xi}_1\dots {\xi}_N\right]\mid e}\right)/{C}_{\left[{\xi}_1\dots {\xi}_N\right]\mid 0} $$

where ξ_n_ is any nucleotide A, T, G or C in the *N*-long word (in the case of tetranucleotides *N* = 4); *C*_[ξ1…ξ*N*]|*obs*_ is the observed count of the word [ξ_1_…ξ_*N*_]; *C*_[ξ1…ξ*N*]|*e*_ is the expected count and *C*_[ξ1…ξ*N*]|0_ is a standard count estimated from the assumption of an equal distribution of words in the sequence: (*C*_[ξ1…ξ*N*]|0_ = *L*_*seq*_ × 4^*-N*^).

Expected counts of words *C*_[ξ1…ξ*N*]|*e*_ were calculated in accordance to the applied normalization scheme. Thus, *C*_[ξ1…ξ*N*]|*e*_ = *C*_[ξ1…ξ*N*]|0_ if OU is not normalized, or *C*_[ξ1…ξ*N*]|*e*_ = *C*_[ξ1…ξ*N*]|*n*_ if OU is normalized by empirical frequencies of shorter words of the length *n* by Markov n-order chain normalization.

Two approaches of normalization by GC content have been exploited where the GC content was calculated either for the sliding window sequence (local normalization) or for the complete reference sequence (generalized normalization).

The distance (*D*) between two OUPs was calculated as the sum of absolute distances between ranks of identical words (*w*, in a total 4^*N*^ different words that is 256 for tetranucleotides) after ordering of the words by Δ_[ξ1…ξ*N*]_ values (eq. 1) in two patterns *i* and *j*:

2 $$ D\left(\%\right)=100\times \frac{\sum \limits_w^{4^N}\left|{\mathit{\operatorname{rank}}}_{w,i}-{\mathit{\operatorname{rank}}}_{w,j}\right|-{D}_{\mathrm{min}}}{D_{\max -{D}_{\mathrm{min}}}} $$

Pattern skew (PS) is a particular case of D where patterns *i* and *j* were calculated for the same DNA molecule but for the direct and reversed strands, respectively. D_max_ = 4^*N*^ × (4^*N*^ – 1)/2 and D_min_ = 0 when calculating a D, or, in the case of PS calculation, D_min_ = 4^*N*^ if *N* is an odd number or D_min_ = 4^*N*^ – 2^*N*^ if *N* is an even number.

Variance of an OU pattern was calculated by the following equation:

3 $$ V=\frac{\sum \limits_w^{4^N}{\varDelta}_w^2}{\left({4}^N-1\right)\times {\sigma}_0} $$

where N is the word length; Δ^2^_*w*_ is the square of a word *w* count deviation (eq. 1); and σ_0_ is the expected standard deviation:

4 $$ {\sigma}_0=\sqrt{0.02+\frac{4^N}{L_{seq}}} $$

where *L*_*seq*_ is the sequence length, and N is the word length.

SWGIS v2.0 calculates two types of variances for the patterns normalized by the GC content of a sliding window (relative variance or RV) and normalized by the GC content of the whole reference sequence (generalized relative variance or GRV). The ratio RV/GRV is then used for GI prediction. These parameters were described in more detail in previous publications [25, 26], where cut-off values for GI predictions were established empirically as the following: D larger than 1.5, PS smaller than 55 and RV/GRV larger than 1.5.

The principle improvement in SWGIS v2.0 was in calculating a reference OUP for a 300 kbp sliding window and recalculating OU for every 100 kbp. The original SWGIS algorithm calculated a reference OUP for an entire bacterial chromosome which is not representative of more heterogeneous chromosomal fragments in larger eukaryotic chromosomes. Also, in SWGIS v2.0, operons of genes encoding ribosomal RNA (*rrn*) were filtered out by high PS values as well as BLASTN against the SILVA database of *rrn* sequences of both eukaryotes and prokaryotes [27].

### Test chromosomes used for artificial insertions of genomic islands

To estimate rates of false positive and false negative GI predictions, chromosomes with artificial GI insertions were created. These test chromosomes were chosen based on a preliminary run of SWGIS v2.0 to identify chromosomes that are naïve (those with no predicted GIs) and those that are non-naïve (containing other predicted GIs). From there, the relevant chromosomes were chosen to adequately represent the different kingdoms available in the database. The following naïve chromosomes were used: *Candida albicans* (NW_139454, NW_139474), *Thalassiosira pseudonana (*NC_012068, NC_012069)*, Torulaspora delbrueckii* (NC_016501, NC_016504)*, Phaeodactylum tricornutum* (NC_011690, NC_011693). The following non-naïve chromosomes were used: *Aspergillus fumigatus* (NC_007194, NC_007194)*, Fusarium oxysporum* (CM000593, CM000594)*, Saccharomyces cerevisiae* (BK006941, BK006942)*, Cryptococcus neoformans* (NC_026749, NC_026750)*, Theileria parva* (NC_007344, NC_007345)*, Plasmodium falciparum* (NC_004329, NC_004330)*, Drosophila melanogaster* (NC_004353, NC_00454)*, Caenorhabditis elegans* (NC_003279, NC_003280).

### False negative estimation of SWGIS v2.0

The Pre_GI [28] database were inspected for GIs that are also contained within the pathogenicity islands database (PAIDB) [29]. Of these, GIs that contained *rrn* sequences were discarded which related to a total of 194 GIs. The sequences of these 194 GIs were inserted into arbitrary locations of different test chromosomes using a randomization simulation. Each simulation inserted a single GI into an arbitrary location, implemented the SWGIS v2.0 algorithm and determined whether the algorithm identified the artificially inserted PAIDB GI. Thus, on each naïve or non-naïve test chromosome, 194 simulations were performed; each simulation with a different PAIDB GI, and a false negative ratio was determined based on the frequency of correctly detecting the inserted PAIDB GI and incorrectly not detecting the inserted PAIDB GI for each test chromosome.

### False positive estimation of SWGIS v2.0

Random genomic fragments, acting as artificial GIs, were arbitrarily transferred between two chromosomes of the same organism with a randomization simulation. The assumption was made that the OU of two chromosomes of the same organism would be similar and detecting an artificially transferred segment can be considered as a false positive. Each simulation transferred a single arbitrary genomic fragment of 28,173 bp, the average length of the 194 GIs from PAIDB used for false negative estimation, from one chromosome to an arbitrary location on the other chromosome, implemented the SWGIS v2.0 algorithm and determined whether the artificially transferred segment was identified as a GI. Specifically, for non-naïve chromosomes, the simulation ensured that the transferred fragments do not already contain a GI(s) and is not inserted in locations that already contain a GI(s). For each test chromosome, a total of 100 simulations were performed; each simulation with a single arbitrary genomic fragment inserted into an arbitrary location and a false positive ratio was determined based on the frequency of correctly not detecting the transferred fragment and incorrectly detecting the transferred fragment.

### Case studies

SWGIS v2.0 was used to identify GIs in the genomes of *Aspergillus fumigatus* and *Drosophila ananassae* as two case studies. Firstly, we compared GIs identified by SWGIS v2.0 in *A. fumigatus* to previously predicted atypical regions in this organism where the variation in OUP across the genome was also used to predict GIs [23]. In the cited work, GI identification was performed by using a parametric method based on local variations of genomic signatures that makes this study useful for benchmarking of SWGIS v2.0. Secondly, we tested for sequence similarity with BLASTN (e-value cut off 1^− 20^) between GIs predicted in *D. ananassae* to the *Wolbachia* endosymbiont (NZ_AAGB00000000) of this species, as previous reports have shown that the entire genome of *Wolbachia* has been transferred to the *D. ananassae* genome [30].

### Genome sequences for database construction

Complete sequences of 1062 chromosomes of 66 eukaryotic organisms were obtained from the RefSeq database in GenBank format using the NCBI FTP server (ftp://ftp.ncbi.nih.gov/genomes/refseq). The RefSeq database was chosen to ensure only high quality assemblies in chromosome format was used and to limit the identification of potential bacterial contaminants, especially in smaller contigs of incompletely sequenced genomes. The EuGI web-resource contains the genome accession numbers of all the sequences used (http://eugi.bi.up.ac.za/eugi_source.php).

### Database software and programming

MySQL package v5.1.73 for Linux was used for database creation. All programming was performed in Python 2.5.

## Results

### Prediction of genomic islands in eukaryotic chromosomes using SWGIS v2.0

The basic principle behind the SWGIS algorithm consisted in a superimposition of values of several statistical parameters calculated for a sliding window. Particularly, GIs were characterized by an increased distance between local tetranucleotide usage patterns and the reference pattern calculated for the complete genome (D-values) and by increased ratio of locally normalized over globally normalized values of local tetranucleotide usage variances (RV/GRV). The approach proved to be useful for distinguishing between horizontally acquired inserts of GI and the core genome sequence, but also this technique allowed discrimination of GIs from other atypical regions such as multiple repeats or loci with an alternative GC content.

**Fig. 1:**
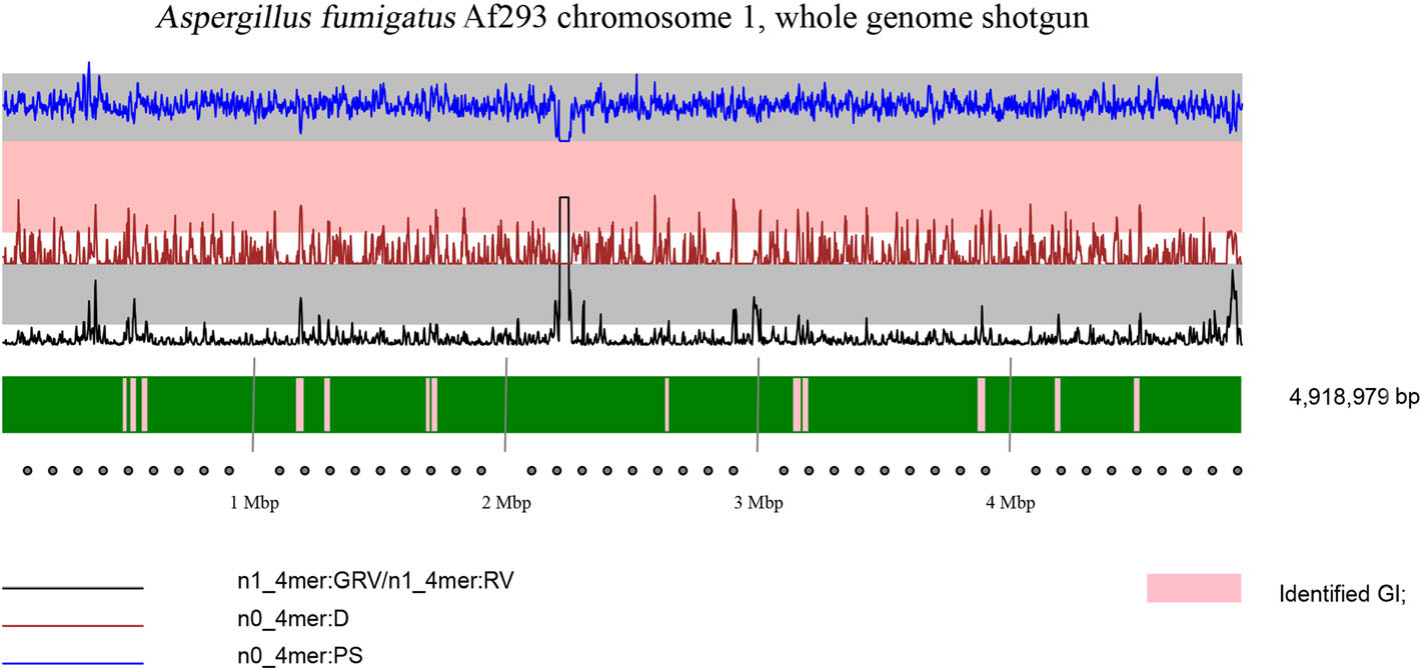
Graphical output of the SWGIS v2.0 algorithm. The SWGIS v2.0 displays GIs (pink blocks) on large linear chromosomes. Several parameters are also shown on the graph: GC-content (black curve); ratio of generalized to local relative variances calculated for tetranucleotide usage patterns normalized by the GC-content (blue curve, n1_4mer:GRV/n1_4mer:RV); distances between not-normalized local tetranucleotide usage pattern and the global pattern calculated for the complete chromosome (red curve, n0_4mer:D); asymmetry between not-normalized tetranucleotide usage patterns calculated for the direct and complement DNA strands (green curve, n0_4mer:PS). Use of these parameters for GI identification and their standard abbreviations were explained in more detail by Ganesan et al. (2008)*.* and on the EuGI website (http://eugi.bi.up.ac.za) [25]

Application of the initial version SWGIS v1.0 designed for prokaryotic organisms cased sometimes an increased false prediction of GIs in eukaryotes. Eukaryotic chromosomes are larger and have a higher intra-sequence heterogeneity compared to prokaryotes and in many cases the OUP calculated for large parts of the same chromosome may differ significantly from local OUP. Calculating a reference OUP of 300 kbp and recalculating OU for every step of 100 kbp in SWGIS 2.0 allowed DNA homogeneity within the reference sliding window and can more accurately predict regions that deviate in its oligonucleotide composition compared to the OUP of the rest of the chromosome. This reduced potential false positive prediction. Another improvement in SWGIS v2.0 was the visual representation of GIs on large linear chromosomes in the form of a scalable vector graphic (Fig. 1). Average computational time for all test chromosomes was approximately 23.66 s/kb on a normal Windows desktop.

### False negative estimation of SWGIS v2.0

Calculating false negative values as the ratio of identifying an artificially inserted GI versus not identifying the imitation GI, showed low values in general. The highest false negative percentage was 42.78% and the lowest was 5.67%. Although a wide range of false negative percentages was observed, the means were 20.8% and 19.4% for naïve (Table 1) and non-naïve (Table 2) chromosomes respectively, with a combined average of 20.1%.

**Table 1:** False positive and false negative values of SWGIS v2.0 calculated for different naïve chromosomes

	False positive ^a^	False negative
*C. albicans* ^NW_139454^	16%	5.67%
*C. albicans* ^NW_139474^	0%	7.73%
*T. pseudonana* ^NC_012068^	0%	30.93%
*T. pseudonana* ^NC_012069^	0%	30.41%
*T. delbrueckii* ^NC_016501^	1%	16.49%
*T. delbrueckii* ^NC_016504^	2%	21.13%
*P. tricornutum* ^NC_011690^	11%	24.74%
*P. tricornutum* ^NC_011693^	1%	29.90%

^a^Random chromosome fragments were moved from the other chromosome of the same organism (e.g. for NW_139454 random fragments were moved from NW_139474 to NW_139454 and vice versa for NW_139474)

**Table 2:** False positive and false negative values of SWGIS v2.0 calculated for different non-naïve chromosomes

	False positive ^a^	False negative
Fungi		
*A. fumigatus* ^NC_007194^	18%	30.93%
*F. oxysporum* ^CM000593^	16%	34.54%
*S. cerevisisae* ^BK006941^	2%	11.34%
*C. neoformans* ^NC_026749^	7%	42.78%
Protozoa		
*T. parva* ^NC_007344^	25%	7.22%
*P. falciparum* ^NC_004329^	22%	5.67%
Invertebrates		
*D. melanogaster* ^NC_004353^	49%	13.40%
*C. elegans* ^NC_003279^	6%	9.28%

^a^Random chromosome fragments were moved from the successive chromosome of the one listed in the table (e.g. for *A. fumigatus* random fragments were moved from NC_007195 to NC_007194, and similarly for the other organisms listed in column one)

### False positive estimation of SWGIS v2.0

Calculating false positive values as the ratio of identifying an artificial inter-chromosomal transfer versus not identifying the insert were low in general except for chromosome 4 of *D. melanogaster* (NC_004353; 49%). False positive values showed a wide range for both naïve (Table 1) and non-naïve (Table 2) chromosomes, with means of 3.88% and 18.13%, respectively, and a combined average of 11.01%. Several naïve sequences had false positives values of 0%.

### Case studies

#### *Aspergillus fumigatus*

**Fig. 2:**
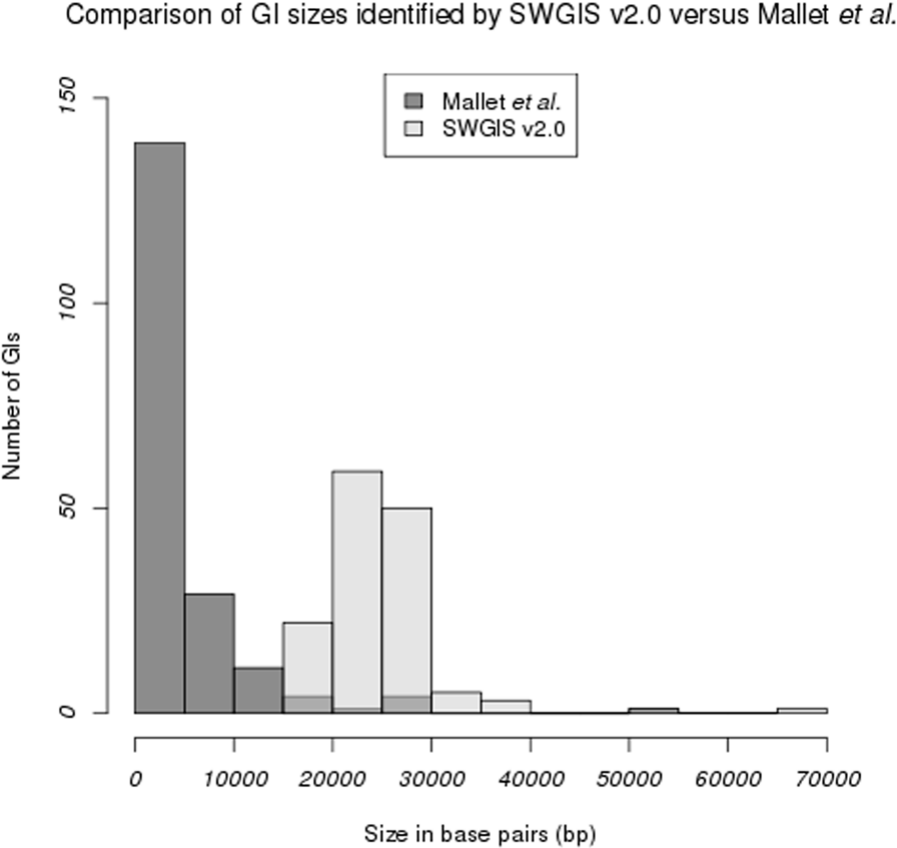
Comparison of GI sizes identified by Mallet et al. and SWGIS v2.0

SWGIS v2.0 identified 141 GIs in the genome of *A. fumigatus*. Previously, 189 atypical regions were identified in this genome (Table 3) [23]. However, several of the previously identified GIs were either small (< 5 kb) or contained *rrn* sequences (Fig. 2). After filtering out small and *rrn* containing GIs from the 189 previously identified GIs (filtering function is embedded in the SWGIS v2.0 algorithm), a subset of only 31 GIs remained for comparison. From this smaller subset of 31 GIs, a total of 18 GIs were confirmed by SWGIS v2.0. Of the remaining 13 GIs of the smaller subset not predicted as GIs by SWGIS v2.0, only five contained MGEs according to the definition used in the original publication [23] and thus represent true positives not identified by SWGIS v2.0. It can therefore be argued that only five GIs were missed by SWGIS v2.0 as false negatives.

**Table 3:** Distribution of GIs identified by SWGIS v2.0 versus Mallet et al.

Chromosome #	1	2	3	4	5	6	7	8	Total
#GIs – Mallet et al.	30	28	28	22	22	31	14	14	189
#GIS – SWGIS v2.0	19	24	26	17	16	20	11	8	141

#### *Drosophila ananassae*

**Fig. 3:**
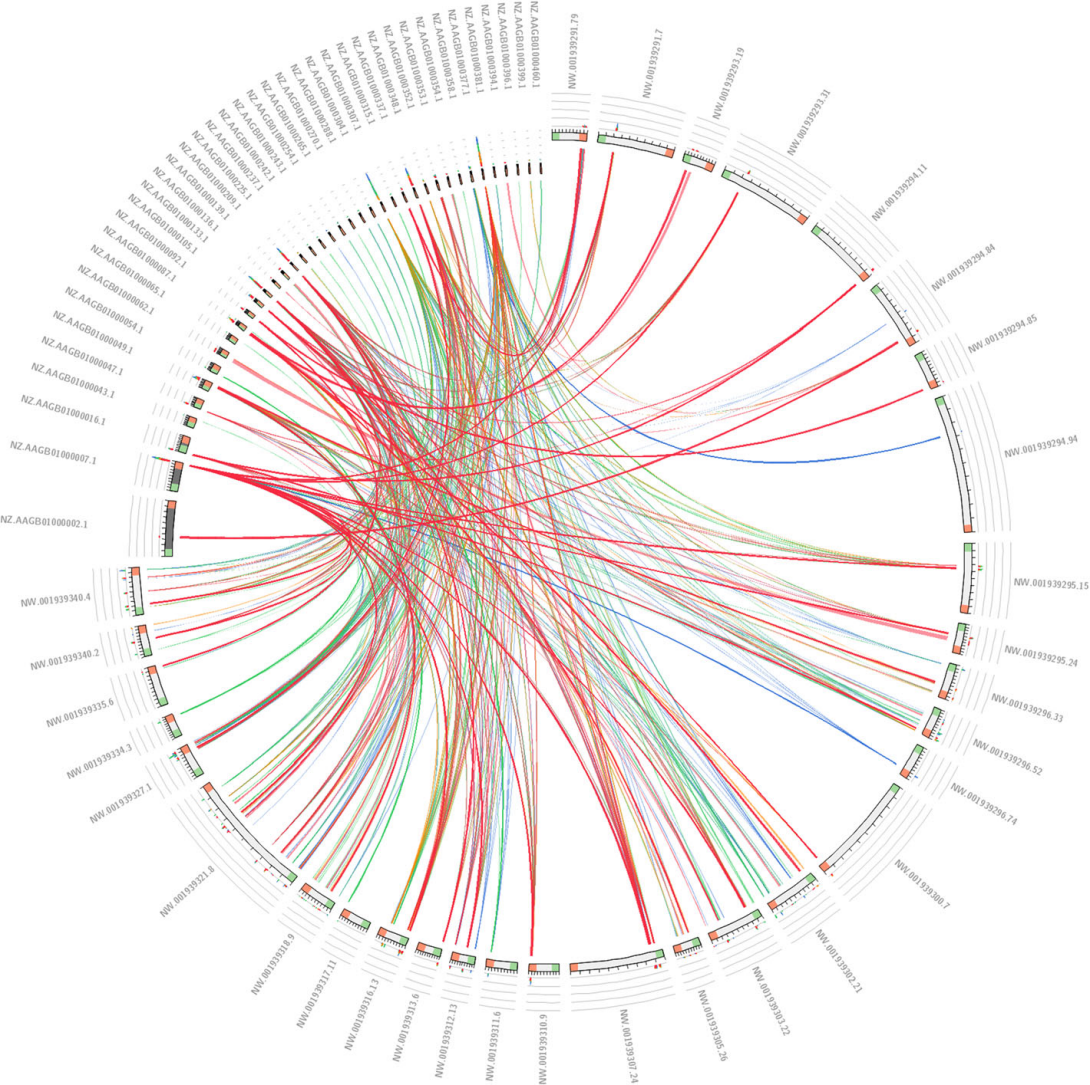
BLASTN alignment of coding GIs identified in *D. ananassae* and the *Wolbachia* endosymbiont of *D. ananassae*. Circoletto [39] was used to visualize sequence similarity of coding GIs of *D. ananassae* and its *Wolbachia* endosymbiont. Scaffolds of *Wolbachia* are represented by their accession numbers from the original GenBank file downloaded from NCBI. Predicted GIs from *D. ananassae* are indicated by the scaffold accession number (e.g. NW.001939327) followed by a number which indicate the number of the GI predicted in the specific scaffold (e.g. NW. 001939327.1). Line colours are indicative of e-values between queries and subjects with red the smallest, orange second, green third and blue the highest e-values

SWGIS v2.0 identified 1288 GIs in the genome of *D. ananassae* (using only scaffolds larger than 300 kbp)*.* Of these, 70 were coding GIs and 1218 were non-coding GIs. BLASTN alignment showed a very high sequence similarity between coding GIs and regions in the genome of the *Wolbachia* endosymbiont of *D. ananassae* (Fig. 3). In some instances, an entire contig of *Wolbachia* showed high sequence similarity to GIs of *D. ananassae*, for example contig NZ_AAGB01000133.1 had an average identity of 93.48% against GIs of *D. ananassae*. Furthermore, many GIs identified in *D. ananassae* constitute fragments of the genome of *Wolbachia* endosymbiont NZ_AAGB00000000.1.

### EuGI database development and analysis

Using SWGIS v2.0 a total of 10,550 GIs were identified in 66 eukaryotic species (Additional file 1: Table S1). All species analysed contained at least one GI, however, 176 fungal chromosomes, 34 protozoan chromosomes and 11 invertebrate chromosomes were naïve, i.e. do not contain any GIs. An overview of GI prediction in different genomes is shown in Table 4.

**Table 4:** GIs predicted in different eukaryotic lineages using SWGIS v2.0

	# species	# chromosomes	# GIs	# coding GIs
Fungi	36	614	3080	2299
Protozoa	22	392	2911	2506
Invertebrate	8	56	4559	494
Total	66	1062	10,550	5299

Identified GIs were subsequently filtered for those that are coding i.e. contained at least one protein coding gene within the GI and those that are non-coding i.e. contained no protein coding genes. Coding GIs were 5299 in total. Large invertebrate genomes contained a large proportion of non-coding GIs (Table 4).

To avoid enriching the database with non-coding elements abundant in eukaryotic genomes, only coding GIs and 28,943 potentially horizontally transferred genes comprising coding GIs were used for further comparative analyses. BLASTN and BLASTP (e-value cut-off 0.01) of 1472 GI nucleotide sequences and 9831 protein translations of constituent genes showed a significant level of sequence similarity against archaeal/bacterial GIs residing in the Pre_GI database [28]. GI sequences, constituent genes, which potentially have been acquired by HGT, and the results of BLAST searches against the Pre_GI database were then used to construct the Eukaryotic Genomic Islands (EuGI) database. The database enables comparison of novel predicted GIs in user data against GIs already predicted in 66 eukaryotic species and stored in the database.

A variety of functions were associated with predicted genes of HGT origin. A total of 17,715 genes were either hypothetical or uncharacterized in their annotation. Genes encoding for different tRNA molecules were found in 342 predicted GIs. Other functional classes of GI-associated genes include 406 kinases, 167 transporter proteins, 166 oxidoreductases, 155 phosphatases, 107 peptidases and 77 hydrolases.

### The EuGI web-resource

To easily visualise and browse eukaryotic GIs predicted by SWGIS v2.0 and the involved genes, the EuGI database was made freely available through the EuGI web-resource (http://eugi.bi.up.ac.za). Genomic sequences of GIs in addition to protein sequences of predicted genes can be downloaded through the resource. Different search and browse tools as well as queries with user data are incorporated into the EuGI web-resource.

SWGIS v2.0 was implemented in the web-resource as a eukaryotic GI finder for users to predict GIs in their own data. This tool allows the upload of either genomic sequences in FASTA format or annotated sequences in GenBank format.

## Discussion

In this study, the SWGIS GI predictor was adapted for the use in eukaryotic genomes, termed SWGIS 2.0. The algorithm of SWGIS v2.0 was shown to have acceptable levels of false negative and false positive estimates of 20.1% and 11.01%, respectively. GIs were predicted using SWGIS v2.0 in 66 different fungal, protozoan and invertebrate genomes. All predicted GIs and potentially horizontally transferred genes within these GIs, together with a repertoire of tools for GI identification are also combined in the EuGI database and web-resource (http://eugi.bi.up.ac.za). This provides one of the first comprehensive analytical suites for GI investigation in eukaryotes.

SWGIS v2.0 is one of the first parametric GI predictors for eukaryotes, and the first that has been tested on a broad range of taxa. While several programs exist that apply parametric algorithms on prokaryotic genomes to identify GIs [1, 2, 31], the identification of GIs in eukaryotic genomes are limited to a single parametric GI predictor optimized for eukaryotes [32] and GI studies of *A. fumigatus* [23]. These two studies were applied to smaller subsets of species or focussed on a single species [23, 32]. Furthermore, rates of false positive and false negative GI predictions in eukaryotic genomes were also estimated in this study, but this information is not available for other parametric GI predictors designed for eukaryotes.

A reference OUP is calculated in the SWGIS v2.0 algorithm for a large (300 kbp) sliding window of the chromosome and recalculated for each step of 100 kbp. OUPs have been shown to be constant genomic signatures and characteristic for whole bacterial chromosomes except for HGT acquisitions, clusters of genes for ribosomal RNA and some other minor loci [26]. However, variations of OUPs across eukaryotic chromosomes have not been explored on a large set of different species, as was done here. The sliding window optimization of the SWGIS program [15] in SWGIS v2.0 accounts for the sequence heterogeneity within eukaryotic chromosomes and allowed the algorithm to distinguish between GIs and other atypical regions.

The rate of false positive predictions in SWGIS v2.0 was reduced by embedded filtering algorithms including filtering of loci containing *rrn* genes and those smaller than 5 kbp. These filters are commonly used to limit false positive prediction in prokaryotic GIs [26, 31]. Accounting for different OUP statistical parameters in SWGIS v2.0 allows distinguishing GIs from multiple other genomic loci with alternative OUPs, i.e. non-coding repeat elements or intergenic regions that reduces false positive predictions.

False positive and false negative values for SWGIS v2.0 are comparable to those from common prokaryotic GI predictors [2]. For example, a comparison between four routinely used prokaryotic GI predictors, SIGI-HMM, PAI-IDA, Centroid and Alien_Hunter (IVOM) showed ranges of 23% - 72% and 8% - 62% for sensitivity and specificity, respectively [2]. The false negative average calculated for SWGIS v2.0 (20.1%) indicates a better sensitivity of the program than those reported for above mentioned GI predictors. The false positive average calculated for SWGIS v2.0 (11%) is within the specificity range reported for these prokaryotic GI predictors.

The identification of GIs with SWGIS v2.0 can alleviate the task of searching through an entire genome for HGT events by predicting inserts of GIs comprising functional genes. Phylogenetic incongruence between gene and species trees is often considered as the ‘golden standard’ for confirming HGT in eukaryotes and is more broadly used to investigate HGT in eukaryotes [5, 9]. Relying solely on phylogenetics to identify GIs have also been shown to underestimate the rate of HGT events in eukaryotes and can consequently result in high frequencies of false negatives [19]. The reason for this is that the sensitivity of these methods is limited by availability of genome sequences of putative ‘GI donor’ organisms in public databases, which won’t be fully comprehensive in the near future. Contrary, parametric OUP based methods allow GI prediction without need for any reference genome that is an important concept in prokaryotic HGT studies [2, 15, 31]. Although downstream phylogenetic analyses remain critical, screening a genome using SWGIS v2.0 to identify GIs can provide a focus for further phylogenetic and functional annotation studies.

The web-accessible EuGI database is the first of its kind resource for eukaryotic GIs. Several online databases of GIs in prokaryotes exist [28, 29, 33], but none for eukaryotes. The EuGI web-resource allows easy and user-friendly browsing of GIs currently residing in the EuGI database through the web-interface. The SWGIS v2.0 program forming part of the EuGI web-resource enables users to easily predict GIs without having to download and compile the program code.

A wide range of functions was associated with genes within identified GIs. tRNA genes have been described to act as integration sites for foreign DNA and can be used as supporting evidence for GI identification [2, 34, 35]. Identifying a high frequency of tRNA genes associated with GIs predicted by SWGIS v2.0 therefore demonstrates the ability of the program to identify atypical regions in large genomes. Versatile functional classes of genes, such as kinases, oxidoreductases, phosphatases, peptidases and hydrolases, were associated with GIs identified in this study. Several of these functions have been shown in other studies to be associated with HGTs [10, 21, 23]. This suggests that HGT potentially contributed to the evolutionary adaptation of eukaryotic organisms studied here [36]. Comparative and phylogenetic studies of all horizontally acquired genes remains critical to conclude that these are indeed true positive HGT events [2]. The results can, however, now serve as a benchmark for further functional characterization and to study the role of HGT in the genome evolution of different eukaryotic species more extensively.

The SWGIS v2.0 program was applied to the genomes of *A. fumigatus* and *D. ananassae*, where HGT have been shown to constitute to both these organisms [23, 30]. Previously predicted GIs in *A. fumigatus* is the only report for horizontally acquired genomic loci in a eukaryotic organism [23]. We were able to filter out several GIs previously predicted in *A. fumigatus* and reduce the set used for comparison against GIs predicted by SWGIS v2.0, because previous reports showed that GIs containing *rrn* genes were most likely false positives and those smaller than 5 kbp were statistically unreliable for the used method [2, 26]. A significant overlap existed between GIs predicted by SWGIS v2.0 and those previously predicted after applying the described filtering. SWGIS v2.0 therefore proved to be more consistent for GI prediction in *A. fumigatus* [2].

The sequence similarity between GIs predicted by SWGIS v2.0 in *D. ananassae* and the *Wolbachia* endosymbiont of this organism confirms the high frequency of DNA exchange between these organisms. This is consistent with previous studies that reported that the entire *Wolbachia* genome had been horizontally transferred to the nuclear genome of *D. ananassae* [30]. Endosymbiont-to-host HGT can be frequent and is a well-studied class of gene exchange in eukaryotes [30, 37, 38]. The results of this study supports the conclusion that horizontally transferred genomic segments can successfully be identified using a parametric algorithm such as SWGIS v2.0.

## Conclusions

Here we reported one of the first GI predictors for eukaryotes, SWGIS v2.0, which used a modified version of the SWGIS algorithm. Importantly, we reported the first estimates for predicting false positive and false negative GIs for a parametric GI search tool tested on eukaryotes. The results showed that SWGIS v2.0 performed well compared to common prokaryotic GI prediction tools. This tool, along with the EuGI database constructed by applying the SWGIS v2.0 algorithm on 66 different eukaryotic species and implementing it on the EuGI web-resource, provide the first comprehensive resource for GI prediction and comparison in higher organisms. Using the SWGIS v2.0 tool along with the EuGI web-resource will greatly aid in studies focussed on analysing the flow of genetic material across taxonomic boundaries. Ultimately, studying of horizontally acquired genes in this manner can contribute to the many unanswered questions about how HGT has influenced the evolution of eukaryotes.

## Additional file

Additional file 1: **Table S1.** Eukaryotic organisms used for database construction by GI prediction with SWGIS v2.0. The table presents all fungal, protozoan and invertebrate species that were used for GI prediction in this study along with the amount of GIs predicted in each species and the amount of genes retained within these GIs. (DOCX 17 kb)
