# Supplementary material for: EuGI: a novel resource for studying genomic islands to facilitate horizontal gene transfer detection in eukaryotes
Source: BMC Genomics. 2018 May 3;19:323. doi: 10.1186/s12864-018-4724-8 (PMC5934851; doi:10.1186/s12864-018-4724-8)
Supplement: Supplementary file 1 — Table S1. Eukaryotic organisms used for database construction by GI prediction with SWGIS v2.0. The table presents all fungal, protozoan and invertebrate species that were used for GI prediction in this study along with the amount of GIs predicted in each species and the amount of genes retained within these GIs. (DOCX 17 kb) [file 12864_2018_4724_MOESM1_ESM.docx]

| Table S1: Eukaryotic organisms used for database construction by GI prediction with SWGIS v2.0 | | |
| --- | --- | --- |
|  | # coding GIs | # genes |
| **Fungi** |  |  |
| *Ashbya gossypii* | 126 | 1474 |
| *Aspergillus fumigatus* | 100 | 258 |
| *Aspergillus nidulans* | 47 | 70 |
| *Candida albicans* | 69 | 1085 |
| *Candida dubliniensis* | 69 | 604 |
| *Candida glabrata* | 126 | 1352 |
| *Candida orthopsilosis* | 18 | 152 |
| *Coprinopsis cinerea* | 65 | 103 |
| *Cryptococcus gattii* | 20 | 25 |
| *Cryptococcus neoformans* | 11 | 12 |
| *Debaryomyces hansenii* | 85 | 877 |
| *Encephalitozoon cuniculi* | 18 | 268 |
| *Encephalitozoon hellem* | 19 | 288 |
| *Encephalitozoon intestinalis* | 14 | 257 |
| *Eremothecium cymbalariae* | 70 | 733 |
| *Fusarium oxysporum* | 86 | 175 |
| *Gibberella zeae* | 23 | 37 |
| *Kazachstania africana* | 35 | 304 |
| *Kluyveromyces lactis* | 13 | 118 |
| *Kluyveromyces thermotolerans* | 118 | 1394 |
| *Komagataella phaffii* | 3 | 26 |
| *Magnaporthe oryzae* | 69 | 119 |
| *Millerozyma farinosa* | 200 | 2251 |
| *Myceliophthora thermophila* | 52 | 67 |
| *Naumovozyma castellii* | 46 | 500 |
| *Naumovozyma dairenensis* | 88 | 758 |
| *Nectria haematococca* | 62 | 116 |
| *Podospora anserina* | 151 | 355 |
| *Saccharomyces cerevisiae* | 76 | 680 |
| *Scheffersomyces stipitis* | 29 | 126 |
| *Schizosaccharomyces pombe* | 22 | 28 |
| *Tetrapisispora blattae* | 132 | 1093 |
| *Tetrapisispora phaffii* | 114 | 1196 |
| *Thielavia terrestris* | 102 | 138 |
| *Torulaspora delbrueckii* | 11 | 144 |
| *Ustilago maydis* | 10 | 26 |
| **Protozoa** |  |  |
| *Babesia bovis* | 47 | 144 |
| *Babesia microti* | 51 | 220 |
| *Cryptosporidium hominis* | 2 | 21 |
| *Cryptosporidium parvum* | 71 | 646 |
| *Dictyostelium discoideum* | 189 | 696 |
| *Leishmania braziliensis* | 174 | 968 |
| *Leishmania donovani* | 204 | 1032 |
| *Leishmania infantum* | 155 | 860 |
| *Leishmania major* | 157 | 912 |
| *Leishmania mexicana* | 168 | 961 |
| *Leishmania panamensis* | 180 | 932 |
| *Neospora caninum* | 117 | 175 |
| *Phaeodactylum tricornutum* | 54 | 146 |
| *Plasmodium cynomolgi* | 138 | 261 |
| *Plasmodium falciparum* | 178 | 424 |
| *Plasmodium knowlesi* | 136 | 300 |
| *Plasmodium vivax* | 140 | 255 |
| *Thalassiosira pseudonana* | 10 | 30 |
| *Theileria annulata* | 78 | 253 |
| *Theileria orientalis* | 78 | 216 |
| *Theileria parva* | 63 | 189 |
| *Trypanosoma brucei* | 116 | 1088 |
| **Invertebrates** |  |  |
| *Anopheles gambiae* | 228 | 296 |
| *Caenorhabditis briggsae* | 31 | 46 |
| *Caenorhabditis elegans* | 98 | 454 |
| *Ciona intestinalis* | 16 | 47 |
| *Drosophila melanogaster* | 46 | 53 |
| *Drosophila pseudoobscura* | 11 | 13 |
| *Drosophila yakuba* | 28 | 44 |
| *Nasonia vitripennis* | 36 | 52 |
| Total | 5299 | 28943 |
